# Recruiting and Training Junior Medical Students as Basic Life Support Instructors for Primary School Students: Prospective Pilot Implementation Study

**DOI:** 10.2196/100385

**Published:** 2026-09-03

**Authors:** Amanta Nasution, Eric Golay, Tara Herren, Emma Hochstrasser, Laure Scherer, Clara Christin, Remi Girerd, Department of Public Instruction Consortium, Marie-Claude Audétat, Noëlle Junod Perron, Simon Regard, Mélanie Suppan, Laurent Suppan

**Affiliations:** 1 Department of anaesthesiology, pharmacology, intensive care and emergency medicine, Faculty of Medicine, University of Geneva, Rue Gabrielle-Perret-Gentil 4Geneva, 1205, Switzerland, +41 786676933; 2 Department of Acute Care Medicine, Division of Emergency Medicine, University Hospital of Geneva, Geneva, Switzerland; 3 Unit of Development and Research in Medical Education, Faculty of Medicine, University of Geneva, Geneva, Switzerland; 4 University Hospital of La Réunion, South Site, La Réunion, France; 5 See Acknowledgements; 6 Cantonal Physician Division, Cantonal Health Office, Geneva, Switzerland; 7 Department of Acute Care Medicine, Division of Anaesthesiology, University Hospital of Geneva, Geneva, Switzerland

**Keywords:** Kids Save Lives, basic life support, instructor training, medical education, undergraduate medical education, medical students, primary school students, primary school, out-of-hospital cardiac arrest, cardiopulmonary resuscitation, implementation, pilot study

## Abstract

**Background:**

Basic life support (BLS) significantly improves survival and neurological outcomes after out-of-hospital cardiac arrest (OHCA). However, bystander cardiopulmonary resuscitation (CPR) rates vary widely worldwide, reaching only 40% in Geneva, Switzerland. The European Resuscitation Council’s “Kids Save Lives” statement advocates for integrating BLS education into mandatory school curricula to improve bystander CPR rates, and thus OHCA outcomes. Training medical students as BLS instructors could help address the shortage of qualified instructors needed to implement school-based BLS programs in primary schools.

**Objective:**

The primary aim of this pilot study was to assess the feasibility of involving medical students as BLS instructors for primary school classes and to evaluate the impact of their teaching on primary school students’ immediate knowledge acquisition and self-confidence.

**Methods:**

This pilot implementation study was conducted in Geneva, Switzerland. Second- and third-year medical students were presented the project during their emergency skills training and invited to complete an online questionnaire. Sixteen students were selected and underwent instructor training, enabling them to deliver a BLS course in a primary school class. Participating seventh-grade primary school students completed a questionnaire designed to assess immediate knowledge acquisition and confidence. The primary outcome was the feasibility of involving medical students as BLS instructors, including the proportion of medical students willing to become BLS instructors, the number trained and certified, and the number of BLS courses delivered. Secondary outcomes included children’s mean knowledge score and confidence in calling the emergency phone number. Predictors of willingness, and of knowledge and confidence, were also examined.

**Results:**

Among 325 eligible students, 107 (32.9%) completed the questionnaire, and 87 (26.8% of all eligible students; 81.3% of respondents) expressed willingness to fully participate in this program. The 16 selected students successfully completed the instructor training and delivered courses in 16 primary school classes. No significant predictors of willingness were identified. A total of 295 (81.7%) of 361 children completed the postcourse questionnaires. Knowledge acquisition was high, with a mean score of 5.4 (SD 1.1) out of 7. High proportions of correct responses were observed for most BLS concepts, except for breathing assessment and recovery position indications. Overall, 78% (230/295) of children reported confidence in calling emergency services. No significant predictors of knowledge or confidence were found.

**Conclusions:**

Medical students can effectively deliver BLS training to primary school students and may represent a useful workforce for school-based BLS programs. However, the low response rate introduces a selection bias, as many students did not respond and the observed willingness may not reflect that of the overall student population. Sustainable scale-up will require involving other instructors. Further studies should assess the feasibility of large-scale implementation.

## Introduction

### Background

Cardiovascular disease is a leading cause of death worldwide, and cardiac arrest is frequently caused by coronary obstruction [1,2]. Most cardiac arrests are out-of-hospital cardiac arrests (OHCAs), and their low survival rate remains a major global health issue [3,4]. Overall survival after OHCA is around 10% in Europe, with rates varying between 7.6% and 20.4% between countries [4]. These differences can be explained by variations in basic life support (BLS) delivery, which improves the survival and neurological outcomes after OHCA [4-7]. Bystander-initiated cardiopulmonary resuscitation (CPR) is of particular importance and can double to triple survival to hospital discharge; conversely, there is a 10% reduction of survival probability for each minute without CPR. Bystander-initiated CPR is also essential in reducing cerebral ischemia time and minimizing the risk of irreversible brain damage [3,5,8]. Despite these benefits and even though most OHCAs are witnessed, BLS maneuvers are infrequently performed by bystanders, with rates varying widely worldwide and ranging from 19.1% to 79.0% [4,9]. Since the professional rescuers’ response time is rarely less than 10 minutes, achieving survival with good neurological outcomes is thus limited without bystanders’ contribution [4,10,11].

### Enhancing CPR Awareness Through Mandatory School Programs

Young people might represent a promising target group for improving the prognosis after OHCA. As they tend to be more responsive to instructions and learn more easily to help others, regular first aid training at a young age can promote long-term retention of BLS skills and knowledge [12,13]. In Scandinavian countries, where CPR training is mandatory in public schools, bystander CPR rates are high, and survival outcomes are better compared to those in other European countries [4,14,15]. Across Europe, only 8% of patients with OHCA survive to hospital discharge [16]. In Denmark, mandatory school programs implemented between 2001 and 2010 significantly contributed to raising awareness, doubling the rate of bystander-initiated CPR (from 21.1% to 44.9%) and tripling the 1-year survival rates after OHCA (from 2.9% to 10.2%) [14]. These encouraging results led to the launch of “Kids Save Lives” campaigns by several international resuscitation organizations, including the International Liaison Committee on Resuscitation (ILCOR) and the European Resuscitation Council (ERC). The “Kids Save Lives” statement recommends that health ministries integrate 2 hours per year of CPR awareness, starting at the age of 12, into mandatory school programs [12]. CPR programs in schools could increase CPR awareness among primary school students, but also among their parents. This “Kids Save Lives” statement recommends that children teach CPR to at least 10 relatives within 2 weeks [12]. In practice, a Norwegian study found that high school students trained an average of 2.8 people each at home, 43% of whom were more than 50 years old [17]. Furthermore, CPR school programs could also ensure that CPR awareness reaches all social classes, including those most at risk but with the least access to training [15]. This initiative enhances overall community preparedness for emergency situations by encouraging families to discuss OHCA and BLS maneuvers, fostering a culture of emergency preparedness.

### CPR Awareness in Geneva, Switzerland

In Switzerland, survival to hospital discharge after OHCA varies between 10% and 17% [1,3]. Although a first aid course certificate is required for obtaining a driver’s license in this country, bystander CPR rates and survival outcomes are not as high as might be expected, given that 78% of adults have undergone first aid courses [18]. This may be explained by a rapid decline in confidence in their ability to perform CPR after completing a CPR training course and the lack of mandatory CPR course refreshers for adults [18]. In contrast with the 2 hours per year recommended from age 12, primary school students in Geneva receive a single 2-hour CPR course in 10th grade (ie, ages 13‐15 years).

The Department of Public Instruction (DPI) aims to develop a longitudinal first aid educational pathway across mandatory schools, building on the existing 10th-grade course, by adding a BLS course in all seventh-grade classes (approximately 310 per year) and all 11th-grade classes. At this stage, 10th-grade first aid awareness courses are delivered by physical education teachers and school nurses who are required to complete BLS refresher courses on a regular basis. However, several obstacles hinder this initiative, such as a lack of instructors [18]. To address this shortage of qualified instructors, recruiting medical students could be a solution, given their numbers and their effectiveness in teaching first aid courses to primary school students [19]. Numerous studies demonstrate that medical student instructors are as effective in teaching first aid courses as other instructors, such as physicians, nurses, and physical education teachers [20,21].

We hypothesized that better implementation of BLS instruction programs in public schools in the state of Geneva may increase widespread CPR awareness among children and their relatives. In the long term, this could increase the rate of bystander-initiated CPR and the 30-day survival after OHCA, as suggested by Wissenberg et al [14]. The primary aim of this study was to assess the feasibility of involving medical students as BLS instructors as a first step toward developing a longitudinal first aid training program in mandatory public schools in Geneva. Feasibility was assessed through medical students’ willingness to become BLS instructors and to deliver BLS courses in primary school classes and through the training of a selection of these students as instructors. The second aim was to evaluate the impact of the BLS course on primary school students’ immediate knowledge and self-confidence.

These 2 aims are complementary: the first establishes whether medical students as workforce can be recruited and trained, while the second verifies whether the courses they deliver are effective for the target population. Demonstrating that trained medical students can produce immediate knowledge gains among primary school students justifies considering them as instructors for a future large-scale program.

## Methods

### Study Design

This was a prospective implementation pilot study which took place at the University of Geneva, Faculty of Medicine (UGFM). An overview of the multiorganizational implementation process, including the actors at each step, is presented in Table 1.

When relevant, methods and results are reported according to the CHERRIES (Checklist for Reporting Results of Internet E-Surveys) and STROBE (Strengthening the Reporting of Observational Studies in Epidemiology) guidelines [22,23].

**Table 1.** Multiorganizational implementation procedure of the school-based BLS program.

Step	Procedure or action	Actors
1	Project design	Investigators: Division of Emergency Medicine (HUG^a^) and DPI^b^
2	Institutional approval, approval of access to medical students’ cohorts	DPI, UGFM^c^ and vice-dean for undergraduate education
3	Ethics submissions	Research education unit (DPI) and CCER^d^
4	Approval of school access, parental-consent procedure and standardized BLS^e^ course plan	Research education unit (DPI) and DPI staff
5	Recruitment of second- and third-year medical students during emergency-related courses (online questionnaire)	Recruiters: physicians, BLS teachers, and senior medical students
6	Selection of 16 students and delivery of SRC-certified BLS instructor course	SRC-certified^f^ instructors and DPI staff
7	Assignment of certified student instructors to seventh-grade classes across schools	DPI staff
8	Delivery of 1.5-hour BLS courses in primary school classes and postcourse questionnaire among primary school students	BLS instructors and DPI staff
9	Data digitization, secure storage (encrypted Swiss server), and analysis	Investigator: Division of Emergency Medicine (HUG)

a HUG: Geneva University Hospitals.

b DPI: Department of Public Instruction.

c UGFM: University of Geneva Faculty of Medicine.

d CCER: Cantonal Commission for Research Ethics on Human Beings.

e BLS: basic life support.

f SRC: Swiss Resuscitation Council.

### Ethical Considerations

A declaration of “no objection” was issued by the Cantonal Commission for Research Ethics on Human Beings (CCER; Req-2024‐01148), as this project was not within the scope of the Swiss Federal Act on Research involving Human Beings [24]. This project was also approved by the DPI, its research education unit, and the vice dean of undergraduate education at UGFM.

Regarding informed consent, medical students provided electronic informed consent by accessing the questionnaire after reading the confidentiality notice. Primary school students’ participation required both the child’s consent and written parental consent. Regarding privacy and confidentiality, only those who wanted to become BLS instructors and who already had a BLS instructor certification were required to provide their identity for the creation of nominative certificates and contact purposes. A token system was used to maintain the anonymity of respondents who did not want to become BLS instructors; official student lists were provided by the UGFM, and each student registered had a personalized link. All collected data were stored on an encrypted MySQL-compatible database (MariaDB, version 5.5.5; MariaDB Foundation) located on a Swiss server and handled in accordance with the European General Data Protection Regulation [25]. No compensation was provided to any participant, and participation was entirely free of charge.

### Part 1: Medical Students

#### Participants and Setting

Second-year and third-year medical students at UGFM represented our first convenience sample. At UGFM, medical students study a 6-year curriculum. A BLS flipped classroom course, which enables them to obtain a certificate allowing them to enlist as first responders, is mandatory for second-year students and optional for first-year students. Third-year students also follow a Pediatric BLS course and a primary examination course. Additionally, many students may have previously completed a mandatory 10-hour first aid course required to obtain a driver’s license in Switzerland [26].

#### Enrollment of Medical Students

Prior to recruitment, all recruiters were provided with information about the purpose of the study and its necessary details to recruit medical students.

The BLS course teachers—who were either an emergency physician, an anesthesiologist, or an internist—presented the project to the second-year students during their BLS courses. Senior medical students, assuming research and teaching assistant positions for clinical skills, presented the project to the third-year students during their primary examination courses. Students were invited to check their university email box, where they received study information and a URL to access the online questionnaire. Students were informed that they would be granted a BLS instructor certificate upon successful completion of the training program.

The study URL provided led to an introductory page where a confidentiality notice and an electronic consent form were displayed. Accessing the questionnaire was considered as informed consent to participate in the study.

Among those who wanted to participate in our program, 16 medical students were selected by the investigators to attend the BLS instructor training. To maximize the pilot project’s chances of success and support its long-term implementation, these students were chosen according to their prior involvement in first aid awareness events organized by the investigators.

Students successfully completing the BLS instructor course received a certificate that allowed them to register for the BLS courses in primary schools. All remaining students interested and those with a BLS instructor certificate were invited to participate in the broader implementation starting in September 2025.

#### BLS Instructor Course

The instructor course was delivered by a Swiss Resuscitation Council (SRC)–certified instructor (Eric Golay). Two BLS instructor courses were organized considering the academic period for students and the instructor availability. As specified by the SRC guidelines, each course consisted of two 7-hour sessions and was limited to 8 participants. During the course, a DPI staff member (Harmony Deprade) presented the age-appropriate teaching method and the standardized BLS course plan developed by the DPI. Upon successful completion of the instructor course, participants received their SRC certificate, enabling them to register in the BLS courses in primary schools.

#### Questionnaire for Medical Students

The questionnaire comprised two sections: the firstgathered demographic, prior experiences, and perspectives data (Table 2); the second collected identifying information, confirm participants’ interest in becoming BLS instructors, and to verify that they do not already hold a BLS instructor certification (Table 2).

**Table 2.** Demographic and personal data.

Survey page, field, and question	Type of question
Page 1	
Demographic data	
Participants’ age	Open (Regex^a^)
Gender	MCQ^b^
Current medical education year	MCQ
Medical major	MCQ
Page 2	
Prior experiences	
Participation in optional BLS^c^ course in the first year	“Yes” or “No”
First responder	“Yes” or “No”
Prior experience in teaching	“Yes” or “No”
If yes, for how many years	Open (Regex^a^)
Personal interests	
Interest in teaching as a future physician	Likert scale
Interest in teaching in an academic career	Likert scale
Interest in acute medicine	Likert scale
Page 3	
Interest confirmation	
Confirmation of participants’ interest	“Yes” or “No”
Already had BLS instructor certification	“Yes” or “No”
Name and surname	Free text
Email address	Free text

a A Regex validation rule was used to avoid invalid entries.

b MCQ: multiple choice question (only one answer accepted).

c BLS: basic life support.

#### Data Collection

The first registration email was sent to each medical student on the day of their emergency-related training, according to their cohorts’ schedule. A second and last reminder promoting registration was sent to all second- and third-year medical students on March 15, 2025.

#### Web-Based Platform

The questionnaire was developed under the Joomla 5 content management system (Open Source Matters, Inc) and created using the Community Surveys Pro (Shondalai) [27].

#### Outcomes

The primary outcome was the feasibility of involving medical students as BLS instructors through the following implementation measures: (1) the proportion of second- and third-year medical students who expressed willingness to become BLS instructors and to deliver BLS courses in primary schools’ classes, (2) the number of students who successfully completed the SRC-certified instructor course, and (3) the number of BLS courses delivered in primary school classes. We examined the likelihood of becoming an instructor according to demographic characteristics, prior experiences, and personal interests.

#### Statistical Analysis

Data were extracted to CSV files. Stata 18.5 (StataCorp LLC) was used for data curation and statistical analysis. The curated data file is available as Multimedia Appendix 1. Potential duplicate entries and incomplete questionnaires were excluded.

A Likert scale was used with psychometric items from “Totally Disagree” to “Totally Agree.” Numerical values were attributed to each item gathered using Likert scales, with positive values assigned to the answers in favor of the underlying concept of the item. The different proportions are presented using descriptive statistics (frequency and percentage). Multivariable logistic regression models were applied to assess associations between students’ characteristics and their willingness to become instructors.

### Part 2: Primary School Students

#### Participants and Setting

A total of 16 seventh-grade classes (generally aged 10‐11 years), belonging to six different schools, were chosen by the DPI to host 1.5-hour BLS courses. The schools selected represented different socioeconomic backgrounds, including schools from the Priority Education Network, a program targeting socioeconomically disadvantaged school populations. Only those who attended the BLS courses were included in our second convenience sample. The number of primary school students attending the class was reported by schoolteachers to the investigators after each BLS course.

#### Questionnaire for Primary School Students

At the end of each BLS course, primary school students were asked to complete the questionnaire. Age and prior first aid lesson experience were collected (Table 3). The questionnaire included 7 multiple-choice questions corresponding to the key BLS concepts addressed during the BLS course. The questionnaire measured self-perceived confidence in calling the emergency phone number. The questionnaire was presented on a double-sided A4 page (Multimedia Appendix 2).

**Table 3.** Questionnaire for children.

Survey page, field, and question	Type of question
Personal data	
Age	Free text
Previous first aid training experience	”Yes” or “No”
Questions	
Actions to take before providing first aid	MAQ^a^
Swiss official emergency phone number	Free text
Information to provide to the emergency dispatcher	MAQ^a^
How to assess normal breathing	MCQ^b,c^^c^
Indications for placing someone in the recovery position	MCQ^b^
Criteria for recognizing cardiac arrest	MCQ^b^
What to do if you are unsure how to proceed	MAQ^a^
Confidence	
Feeling able in calling the emergency number	“Yes”, “Maybe”, or “No”

a MAQ: multiple answers questionnaire (more than one answer accepted).

b Four statements were presented. Students were asked to answer true or false for each statement.

c MCQ: multiple choice questionnaire.

#### Data Collection

Children’s participation was voluntary and anonymous. All primary school students had the opportunity to complete the questionnaire for pedagogical and equity reasons. Only questionnaires from children who had consented and for whom parental consent had also been obtained were collected by schoolteachers. Accordingly, a parental information and consent procedure was set up. Investigators sent to the children’s parents a letter (Multimedia Appendix 3), including a project presentation and a statement acknowledging that our project was part of a scientific research program. They were asked to complete the detachable bottom of the letter and return it to the schoolteachers. It was specified that answering the questionnaire was intended to reinforce the newly acquired knowledge, according to the “Kids Save Lives” recommendations [28]. However, no evaluation was given. Parental consent was obtained for the use of their child’s data for research purposes.

#### Outcomes

For primary school students, outcomes included the mean questionnaire score obtained by primary school students and the proportion reporting confidence in calling the Swiss official emergency phone number. We also assessed whether higher mean questionnaire scores among primary school students were associated with age, school socioeconomic status, and prior first aid training.

#### Statistical Analysis

Data were extracted to CSV files. Stata 18.5 (StataCorp LLC) was used for data curation and statistical analysis. The curated data file is available as Multimedia Appendix 4.

The different proportions are presented using descriptive statistics (frequency and percentage). Linear regression models were applied to examine associations between children’s characteristics (age, prior experience, and school socioeconomic status) and both BLS knowledge scores and self-reported confidence in calling the Swiss official emergency phone number. Results were reported as mean scores. A *P* value of less than .05 was considered statistically significant.

## Results

### Overview

The results are presented in 2 parts, involving 2 different populations, sets of methods, and outcomes. We first report the findings related to the medical students (Part 1), and then those related to the primary school students (Part 2). To clarify, the scope of each component, their respective populations, interventions, data collection, and outcomes are summarized in Table 4.

**Table 4.** Separation of study components: medical students vs primary school students.

Dimension	Part 1: medical students	Part 2: primary school students
Population	Second- and third-year medical students at UGFM^a^ (325 registered)	Seventh-grade students, ~10‐11 years, 16 classes of 6 schools (361 attended)
Role in study	Potential BLS^b^ instructors	Recipients of the BLS courses
Intervention	SRC-certified^c^ instructor course (2×7-hour sessions)	1.5-hour standardized BLS course delivered by students’ BLS instructors
Data collection	Online questionnaire: demographics, prior experience, interests, willingness	Post-course questionnaire: knowledge and confidence in calling 144^d^
Primary outcome	Proportion willing to become instructors	Mean immediate knowledge score and proportion confident in calling 144
Response	107/325 (32.9%) responded; 87 willing; 16 selected and certified; 16 courses delivered	295/361 questionnaires completed (81.7%)
Main result	No significant predictors of willingness (model *P*=.17); interpret with caution (selection bias)	Mean score 5.4 (SD 1.1) out of 7; 78% confident in calling 144; no significant predictors
Analysis	Multivariable logistic regression	Multivariable linear regression

a UGFM: University of Geneva Faculty of Medicine.

b BLS: basic life support.

c SRC: Swiss Resuscitation Council.

d 144: Swiss emergency phone number.

### Students' Interest in Becoming BLS Instructors and Teaching in Primary Schools

A total of 163 second- and 162 third-year medical students were registered at UGFM at the time this pilot study started (February 2025). By April 1, 2025, 107/325 (32.9%) medical students had completed the questionnaire, of whom 87 (26.8% of all eligible students; 81.3% of respondents) confirmed their interest in becoming BLS instructors and teaching BLS courses in primary schools. A total of 20 students indicated that they did not wish to become BLS instructors; their characteristics are not detailed here but available in Multimedia Appendix 1.

**Figure 1.**
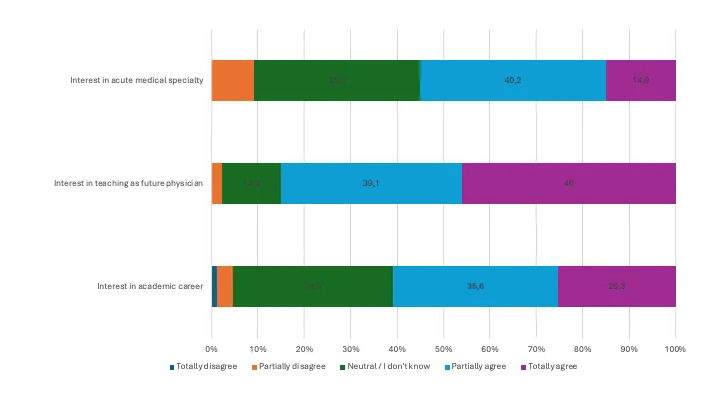
Medical students’ personal interest.

Characteristics of students interested in becoming instructors are detailed in Table 5, and their career interests can be seen in Figure 1. In multivariable logistic regression analysis, none of the included variables, including first responder status, were independently associated with willingness to become a BLS instructor (overall model *P*=.17).

**Table 5.** Characteristics of the students interested in becoming BLS^a^ instructors (n=87).

Characteristics, answers	Values
Gender, n (%)	
Female	51 (58.6)
Male	35 (40.2)
Other	1 (1.2)
Age, mean (SD)	21.2 (1.6)
Bachelor year, n (%)	
Second-year	56 (64.4)
Third-year	31 (35.6)
Major, n (%)	
No specific major	58 (66.7)
Medical education	5 (5.8)
Research	12 (13.8)
Global health and humanitarian	10 (11.5)
Primary care	2 (2.3)
Prior interest in BLS faculty program, n (%)	
Participated in the first-year BLS course program	49 (56.3)
Active as first responder	27 (31.0)
Teaching experience, n (%)	50 (57.5)
Number of years, mean (SD)	1.8 (2.0)

a BLS: basic life support.

All 16 medical students completed the BLS instructor course, and each delivered one BLS course.

### Primary School Students Immediate Post-course Knowledge and Self-Confidence

A total of 361 primary school students attended the BLS courses. By June 14, 2025, 295 of 361 (81.7%) questionnaires had been collected. Table 6 shows the characteristics of the primary school students who completed the questionnaire. Table 7 presents the key BLS concepts retained by primary school students immediately after the BLS course, along with their self-reported confidence in calling the Swiss official emergency phone number (144).

**Table 6.** Characteristics of the participating primary school students (n=295).

Characteristics, answers	Values
Age, mean (SD)	10.8 (0.5)
Prior first aid lesson, n (%)	
Yes	115 (38.9)
No	163 (55.3)
Unknown	17 (5.8)
Education network, n (%)	
Priority	189 (64.1)
Non-priority	106 (35.9)

**Table 7.** Key BLS^a^ concepts retained by children and self-confidence in calling the Swiss official emergency phone number (n=295).

Field, questions	Correct, n (%)	Wrong, n (%)
Knowledge questionnaire		
Actions to take before providing first aid^b^	274 (92.9)	21 (7.1)
Swiss official emergency phone number^c^	284 (96.3)	11 (3.7)
Information to provide to the emergency dispatcher^d^	282 (95.6)	13 (4.4)
How to assess normal breathing^e^	173 (58.6)	122 (41.4)
Indications for using the recovery position^f^	132 (44.7)	163 (55.3)
Criteria for recognizing cardiac arrest^g^	223 (75.6)	72 (24.4)
What to do if unsure as to how to proceed^h^	238 (80.7)	57 (19.3)
Feeling able in calling the emergency phone number, n (%)		
Yes	—^i^	230 (78.0)
Maybe	—	54 (18.3)
No	—	11 (3.7)

a BLS: basic life support.

b Correct answer must include at least “do not put yourself in danger” among other answers.

c Only accepted answer: 144.

d Correct answer must include its position among other answers.

e Correct answer must be a combination of airway opening and checking for breathing by looking, listening, and feeling for 5‐10 seconds.

f Correct answer must be “the person is unconscious but breathing normally.”

g Correct answer must be “the person is unconscious but breathing abnormally.”

h Correct answer must include at least one of the following: starting CPR or calling the emergency number to ask for advice.

i Not applicable.

The mean BLS knowledge score of the children was 5.4 (SD 1.1) out of a maximum of 7 points. In multivariable linear regression analysis (Table 8), none of the included characteristics were significantly associated with total score (overall model *P*=.15; *R*²=0.02). No statistically significant association was found for socioeconomic education network status (β=−0.27; *P*=.06).

**Table 8.** Multivariable linear regression of children’s BLS^a^ knowledge scores (n=295).

Children characteristics	Regression coefficient (95% CI)	*P* value
Socioeconomic education network	−0.27 (−0.55 to 0.01)	.06
Age	−0.09 (−0.35 to 0.15)	.45
Prior first aid experience	−0.13 (−0.41 to 0.13)	.32

a BLS: basic life support.

Following the training, 230 (78%) of 295 primary school students reported feeling confident in calling emergency services. Multivariable linear regression showed that the overall model was statistically significant (*R*²=0.04; *P*=.02), although it explained only a small proportion of the variance in confidence. None of the individual predictors were significantly associated with confidence.

## Discussion

### Principal Findings

The objective of this pilot study was to assess the feasibility of involving medical students as BLS instructors for school-based BLS courses, and to evaluate the impact of these courses on primary school students’ immediate knowledge and self-confidence. These 2 parts of the study are complementary rather than independent: the first demonstrates the feasibility of producing certified instructors, and the second tests whether the courses they deliver achieve their educational goal. We acknowledge that the 2 outcomes are only indirectly related—willingness to teach does not guarantee teaching effectiveness—which is why we evaluated the children’s outcome.

A total of 87 medical students at UGFM expressed willingness to fully participate, exceeding the minimum target of 16 participants for this pilot phase. The findings from primary school students support the immediate effectiveness of this approach, with a satisfactory questionnaire completion rate of 81.7% (295/361) that strengthens the validity of this part, suggesting that medical students can deliver effective BLS courses adapted to this population.

The rationale for school-based BLS training extends beyond the classroom. Its impact should be considered at the population level: by training children, BLS awareness can reach families through vertical transmission, with each child reaching between 1.8 and 4.9 additional individuals [29,30]. Supporting this dissemination effect through tools such as flyers or e-learning could enhance impact. Further research is needed to assess how this initiative influences relatives’ knowledge and engagement in first aid behaviors. This population-level potential is what makes the sustainable delivery of such programs and therefore the availability of instructors, a central issue.

Translating this model into an implementation reaching the approximate 310 targeted seventh-grade classes each year defined by the DPI remains challenging. Despite students’ motivation and the workforce they represent, a student-based model alone is insufficient to reach the targeted number of classes in our context. Nevertheless, our findings support the integration of medical students into future large-scale implementation of BLS courses in seventh-grade primary schools in the state of Geneva. Sustainable “Kids Save Lives” implementation in our context requires moving beyond a student-based model toward an interprofessional model involving other qualified instructors.

### Medical Students' Willingness to Participate in BLS Instruction

Most interested students had attended the optional first-year BLS course, and approximately one-third were already engaged as first responders, suggesting an early interest in emergency care as a determinant of engagement. Nevertheless, in this study, 55.2% (48/87) of students reported interest in acute care specialties. This is consistent with previous studies showing that medical students feel expected to act in emergency situations, although they feel unprepared to face OHCA and do not have much more knowledge than that of the general population [31-33].

Second-year students were more interested in our project than their third-year counterparts. This difference may be explained by the perceived meaningfulness of the teaching activity, as well as by social and contextual factors influencing student engagement.

First, recruiters were different between cohorts, with second-year students introduced to the project by senior physicians and third-year students by senior peers. Third-year students might have been expected to respond more to near peers’ recommendations than to those of senior physicians [34,35]. However, second-year students were more responsive to physicians recruitment interventions. Physicians may be perceived as positive role models for medical students, and their engagement in public health issues may reinforce the associated values, which may influence student motivation through identification with admired figures [36-38]. In addition, given that most students are more oriented toward the clinical field, encouragement from clinicians may further influence students [38]. Moreover, physicians’ awareness of the challenges associated with OHCAs may emphasize the importance of students’ participation, thereby generating interest [37,39].

Timing within the academic year may have also influenced participation. Having recently completed their cardiovascular unit, second-year students may be more aware of cardiac arrest as a public health issue. The cardiovascular unit is a preclinical organ-system module covering mainly basic and pathophysiological sciences, together with epidemiology and clinical skills lectures, but without any clinical placement. At UGFM, neither second- nor third-year students have undertaken in-hospital clinical placements. For second-year students, this limited clinical exposure may make a hands-on community activity such as ours particularly attractive. By contrast, third-year students faced a heavier workload generating frustration, as recently reported in an institutional survey, which may have left them with less availability. Participation in this initiative may therefore have represented an opportunity for more hands-on involvement at a mainly preclinical stage of the curriculum.

Overall, willingness appears to be influenced by early exposure to BLS training and the impact of physicians as role models. These findings suggest that recruitment strategies should target early-year students and involve physicians to optimize engagement. Further research is needed to better understand mechanisms influencing medical students’ engagement in our “Kids Save Lives” initiative.

These findings must, however, be interpreted considering the recruitment strategy and its selection bias. Only 32.9% (107/325) of eligible medical students completed the questionnaire, and respondents were those already interested in teaching and acute care medical specialties. In absolute terms, the 87 students willing to become instructors represent a substantial and useful operational number, well above the needs of this pilot. They correspond to 26.8% of all 325 eligible students (87/325). Because the number of respondents (n=107) was close to the number of interested students (n=87), it is likely that many students who were not interested simply did not complete the questionnaire. Therefore, the willingness proportion describes those who responded to the questionnaire, not the entire second- and third-year medical students’ cohort. Consequently, when generalizing this initiative to a state-wide scale, the true proportion of willing students cannot be extrapolated from the pilot. However, we note that this proportion is expressed relative to all 325 eligible students (87/325, 26.8%), a denominator that already counts nonrespondents as nonwilling. It represents a lower-bound rather than an inflated estimate. A higher response rate, additional recruitment strategies such as more reminders or in-class completion, and the collection of reasons for nonparticipation would strengthen the external validity of this outcome.

Although reasons for nonparticipation were not collected, 2 students who did not enroll provided informative feedback. One student reported a genuine interest in participating, particularly as a future pediatrician, who valued teaching first aid skills to children but was unable to attend because the 4 proposed instructor training days coincided with mandatory classes. The other asked for more information about the BLS courses, notably whether instructors would face a whole class alone or teach in pairs, suggesting that clearer information and teaching in pairs could encourage participation. These comments illustrate that scheduling conflicts and uncertainty about the teaching, rather than a lack of interest, can discourage the participation of motivated students and support the value of collecting reasons for nonparticipation in future phases.

### Immediate Knowledge Acquisition and Confidence in Children Following BLS Training

The second objective was to assess immediate BLS knowledge retention and confidence in calling the Swiss emergency phone number among primary school students following their BLS training. Overall, children achieved high scores across most learning objectives, particularly for the Swiss emergency phone number, information to provide to emergency dispatchers, and appropriate actions when unsure how to proceed. Neither age nor prior first aid training was associated with higher knowledge scores, although both are linked to improved knowledge acquisition [40-42]. The age range was very narrow (mean 10.8, SD 0.5 years) because all primary school students belonged to the same school grade. Given this limited variability, the absence of an association between age and knowledge scores is unsurprising and should not be interpreted as evidence that age has no influence on BLS knowledge acquisition. Assessing such an effect would require a sample with a broader age range. While neither parameter significantly influenced children’s immediate retention of BLS knowledge, this pilot study lacked power to assess the impact of socioeconomic education network status.

These results align with the course objectives defined by the DPI, which emphasize safety, activation of the chain of survival, and initial patient assessment. They are consistent with previous studies showing that children of this age and even younger can correctly provide the emergency phone number and key information following school-based BLS training [13,40,41,43].

Other BLS objectives were also achieved but considered lower priority, as practical maneuvers are emphasized later in 10th grade. Another reason to place the recovery position and CPR as secondary priorities is that evidence indicates that most children do not have sufficient body mass to achieve recommended chest compression depth [28,40,44,45]. Nevertheless, CPR and the recovery position were introduced for familiarization, with the expectation that children will acquire foundations by the time of the 10th-grade refresh. The use of automated external defibrillator (AED) was not included in this pilot due to financial constraints related to future large-scale implementation, although studies indicate that children aged 10‐12 years can use AEDs effectively even months after training [28,40,45-47].

Despite these results, lower scores for certain items suggest that patient assessment may require greater emphasis in future BLS training. Breathing and consciousness assessment should be clarified to improve performance of the recovery position. Evidence in the literature is mixed, likely reflecting heterogeneity of school-based CPR training methods and contexts. While several studies report that children aged 4‐15 years can correctly assess breathing and consciousness and recognize cardiac arrest [28,40,48], other studies report low rates of correct breathing assessment after training among children of similar age [40,49]. Overall, results suggest that patient assessment may not be sufficiently emphasized or clearly conveyed in BLS training.

While immediate knowledge retention was high, the durability of these effects remains uncertain. Previous studies have shown declines in knowledge and skills within months to years after training, although levels remain higher than baseline [50,51]. In contrast, after 3 years of school-based CPR training followed by a pause of 3 years, skills are comparable to 6 years of continuing annual CPR training [52]. In our context, this potential decline of knowledge may be mitigated by refresher BLS sessions implemented in 10th and 11th grade. As evidence remains unclear, further research is needed to evaluate long-term effects of the longitudinal first aid pathway in mandatory public schools in Geneva.

Finally, first aid training focuses on technical BLS skills, with less attention given to misconceptions that may act as barriers to performing first aid. This may partly explain the persistently low rates of bystander CPR attempts, despite ongoing efforts to improve bystander response. Regard et al have shown that confidence among adults to perform first aid declines within 6 months after training [18]. In this study, 78% (230/295) of primary school students reported feeling confident in calling the emergency number immediately after the BLS course, although the durability of this effect remains uncertain. No significant predictors of higher confidence were identified, although trends were observed for younger age and higher socioeconomic schools. Confidence may evolve differently among children and may vary according to age and developmental stage [12]. For example, a recent “Kids Save Lives” study reported higher self-reported willingness to intervene among primary school students compared with secondary school students (138/152, 90.8% vs 45/70, 64.3%) [53]. Understanding how confidence develops during childhood is therefore essential to enhance the effectiveness of BLS courses [54].

Additional factors such as teaching methods, classroom dynamics, or gender may influence outcomes. Gender could not be examined due to restrictions by the DPI. Examining gender at this age would have been of particular interest as previous studies report no significant differences between children of this age group [40]. Studies have found that adult female bystanders have a lower likelihood of providing bystander CPR [55,56]. Whether such patterns already emerge in childhood remains unclear.

### Large-Scale Implementation, Scalability, and Sustainability of a School-Based BLS Program

The DPI of the state of Geneva aims to develop a longitudinal first aid educational pathway across mandatory schools, building on the existing 10th-grade course by adding a BLS course in all seventh-grade classes (approximately 310 per year) and all 11th-grade classes.

Based on this pilot phase, UGFM approved the creation of an optional program embedded in the undergraduate medical curriculum. This initiative is expected to ensure systematic recruitment of students each semester while increasing participation by embedding engagement within formal training. This program consists of 10 mandatory afternoon sessions over one semester. Enrolling 32 students per semester (16 pairs) would allow the delivery of 96 courses per semester, or 192 per academic year. Students will complete an SRC-certified instructor course, emphasizing age-appropriate pedagogical strategies. Instead of attending classes, students’ duties will consist of delivering BLS courses in primary school classes.

This future implementation is grounded in the framework of intervention implementability, including acceptability, feasibility, fidelity, scalability, and sustainability [57].

Acceptability will be reflected in program selection rates and enhanced by embedding teaching activities within formal training, combined with SRC-certified BLS instructor training. This approach may increase its educational relevance and reduce extracurricular burden, identified as a potential participation barrier. Primary school students’ engagement during this pilot phase supports the relevance and accessibility of BLS training at this age.

Feasibility of adding a BLS course in all seventh-grade classes will be supported by the optional program allowing systematic recruitment but will also depend on the coordination of 2 institutions with distinct governance structures, calendars, and priorities. On the university side, UGFM controls the recruitment of medical students and their training as instructors. On the DPI side, responsibilities include access to classrooms, the requirement for DPI staff to attend teaching sessions to ensure quality, the restriction of courses to Thursday afternoons (time dedicated to the future optional program), and the alignment of course content with the school program. Reconciling these constraints requires a formalized governance structure, shared planning, and a designated coordination role able to arbitrate scheduling and quality-assurance decisions. The absence of such a structure constitutes a key organizational risk for long-term scalability.

Fidelity will be supported through standardized training for both primary school students and medical students, including instructor training, and the BLS course plan. Students will teach in pairs, allowing peer support and reducing variability. In addition, systematic feedback provided by DPI staff members after each course will ensure consistency across groups and alignment with pedagogical objectives.

While the optional program is expected to increase teaching capacity, it does not cover the target of reaching all 310 seventh-grade classes. Ensuring full coverage will require expanding the pool of instructors. Involving other health care trainees represents a promising strategy. In Geneva, paramedic training already includes an SRC-certified BLS instructor training and mandatory teaching activities to be completed. No equivalent program currently exists for nursing students. Beyond health care trainees, primary school teachers have been identified as effective BLS instructors according to the “Kids Save Lives” recommendation [28]. They may represent the most cost-effective strategy for delivering annual BLS awareness courses due to their pedagogical expertise and direct access to all classes [28]. However, DPI staff members reported concerns regarding workload constraints and their ability to deliver BLS instruction, which contrasts with evidence supporting their effectiveness and recommending BLS instructor training during their initial university education [28,47,58].

Finally, sustainability will depend on continued acceptability among students, stable DPI support, and the formalized coordination reconciling the institutional constraints of both partners. Embedding this initiative within the UGFM curriculum provides a strong foundation by ensuring recruitment and training of new instructors, but maintaining engagement among successive students’ cohorts, ensuring consistent teaching quality over time, and identifying additional qualified human resources will remain essential. Achieving sustainable large-scale deployment of this “Kids Save Lives” initiative will therefore require a coordinated system-level approach integrating multiple stakeholders.

### Educational and Professional Development Benefits of Medical Students' Involvement in BLS Training

Beyond public health objectives, integrating this initiative into the curriculum aligns with several key educational and professional development goals of the UGFM [59].

First, this program provides an opportunity to develop pedagogical training and teaching opportunities during undergraduate medical training. Currently, opportunities for undergraduate students remain limited, despite expectations that residents rapidly assume teaching responsibilities toward medical students. In this study, 57.5% (50/87) of students already had prior teaching experience, suggesting an existing interest. This initiative may help respond to this interest and contribute to the longitudinal medical education pathway.

Second, numerous studies evaluating similar initiatives report benefits in professional competencies, particularly in BLS knowledge and skills [19,60]. This is particularly relevant considering concerns regarding insufficient BLS skills and knowledge among European medical students according to the European Resuscitation Council [61], including UGFM students [33]. In addition, a study conducted at UGFM showed that senior students approaching graduation reported that the curriculum did not provide them with enough knowledge and skills regarding resuscitation [62]. Overall, enhancing BLS training may address both educational gaps and public health priorities.

Third, our findings highlight the value of students’ engagement in professional roles alongside formal training. By acting as health educators, students engage in professional activities aligned with their future roles. This initiative aligns with the concept of medical students as health coaches, contributing not only to their own learning but also to public health objectives [37]. Their involvement may change their perception of their role within the health care system, even prior to clinical clerkships.

This initiative emerges in a context where an institutional survey conducted at UGFM in April 2024 reported limited real-life practice learning experiences among junior students, leaving many struggling to find meaning in their studies and develop relevant representations of their profession. Such limitations may hinder the development of professional identity (PI), a key outcome of medical education. PI development is acknowledged as a complex process involving internalization of professional values and norms [63]. This process enables medical students to identify themselves as future physicians [64,65]. Among factors shaping PI development, social interactions—with peers, patients, and health care professionals—play an essential role in influencing PI development through commitment to norms and expectation of their future profession [64-66]. Consequently, opportunities for such interactions during undergraduate medical school, whether through clinical or community-based experiences, are essential. Our initiative may provide such opportunities and an early entry point into a community of practice. Acting as instructors may foster students’ sense of responsibility, usefulness, and belonging to the professional community, while supporting the development of a professional posture and confidence as future physicians. In addition, this program may also enhance public recognition of students’ roles within the health care system. A qualitative approach is required to further explore how participation in this “Kids Save Lives” initiative influences professional identity formation [67].

### Limitations

Several limitations must be acknowledged. First, the recruitment strategy may not effectively reach eligible students, as a proportion of second- and third-year students may not attend recruitment interventions in person. These students were reached through email recruitment, potentially reducing their likelihood of participation. In addition, students’ academic commitments are expected to remain the priority, although the instructor training and BLS courses in classes were scheduled outside examination periods. This is consistent with the feedback of the motivated student who declined to participate because the proposed training days overlapped with mandatory classes.

Second, the quality of the materials, including the recruitment email and the questionnaires, could also influence participation. Easy-to-use and concise materials are likely to promote participation. In our case, the recruitment email and the questionnaire, when taken together, were probably too long, which may have discouraged completion, particularly among students who were not already interested in becoming instructors.

Third, participation in this pilot phase relied on voluntary engagement in an extracurricular initiative. It introduces a potential selection bias toward motivated students, particularly those already interested in emergency medicine. The interpretation and consequences of this bias for future large-scale recruitment are discussed above.

Fourth, this pilot phase involved a limited number of students compared to the full-scale deployment including 310 targeted classes. It may therefore not fully capture operational constraints, and its results may not be transposable to a state-wide implementation.

Fifth, this study was conducted within a specific context in the state of Geneva, which may limit the generalizability of findings to other settings with different health authorities, constraints, resources, and curriculum. However, many places face similar challenges, such as lack of human resources, insufficient BLS competencies among the general population and children, and growing expectations for integration of first aid training in schools. Concretely, the instructor training, BLS course plan, and the BLS knowledge questionnaire for primary school students may be used in other contexts. More specifically, the transferability of the model depends on the local coordination between academic and educational authorities. Institutions without comparable partnerships may face different organizational constraints.

Sixth, the sample sizes were limited, which reduced the statistical power of the multivariable analyses. The absence of statistically significant predictors of willingness to become an instructor, and of knowledge or confidence among children, should not be interpreted as evidence that no such associations exist, but rather as a possible consequence of insufficient power to detect them. It is also possible that the questionnaires did not capture the factors that genuinely influence students’ willingness or children’s outcomes.

Finally, this pilot study focused on short-term outcomes, including immediate knowledge retention and self-reported confidence among primary school students, and did not assess long-term retention of BLS competencies.

### Conclusions

This pilot study shows that medical students are willing and able to deliver BLS courses to primary school students, with enough volunteers to support the pilot, and may represent a useful workforce for school-based BLS programs. This willingness should nonetheless be interpreted with caution, as the low response rate introduces a selection bias and limits the validity of this outcome, as many students did not respond and the willingness observed may not reflect that of the overall student population.

Sustainable implementation of BLS courses for all seventh-grade classes in our context requires moving from a medical student-based model to a broader approach involving other instructors. Beyond its public health impact, this approach offers a meaningful community-based opportunity for medical students and may support their professional identity formation. Based on these pilot findings, the next phase will focus on scaling up and refining the implementation strategy to ensure long-term feasibility and integration within the mandatory school curriculum.

## Supplementary material

10.2196/100385 Multimedia Appendix 1 Curated dataset of medical students used in the study.

10.2196/100385 Multimedia Appendix 2 Basic life support knowledge questionnaire for children used to assess knowledge and confidence among schoolchildren.

10.2196/100385 Multimedia Appendix 3 Letter sent to parents to request consent.

10.2196/100385 Multimedia Appendix 4 Curated dataset of schoolchildren used in the study.

## Abbreviations

AED: automated external defibrillator
BLS: basic life support
CCER: Cantonal Commission for Research Ethics on Human Beings
CHERRIES: Checklist for Reporting Results of Internet E-Surveys
CPR: cardiopulmonary resuscitation
DPI: Department of Public Instruction
ERC: European Resuscitation Council
ILCOR: International Liaison Committee on Resuscitation
OHCA: out-of-hospital cardiac arrest
PI: professional identity
SRC: Swiss Resuscitation Council
STROBE: Strengthening the Reporting of Observational Studies in Epidemiology
UGFM: University of Geneva Faculty of Medicine
