# Supplementary material for: Recruiting and Training Junior Medical Students as Basic Life Support Instructors for Primary School Students: Prospective Pilot Implementation Study
Source: JMIR Form Res. 2026 Sep 3;10:e100385. doi: 10.2196/100385 (PMC13541228; doi:10.2196/100385)
Supplement: Supplementary file 1 [file formative-v10-e100385-s002.docx]

**Ce que tu as appris sur les gestes qui sauvent**

**Quel âge as-tu ?** ___________

**Avais-tu déjà vu ces notions de premiers secours dans le passé** :  Oui	Non

**1. Entoure la bonne réponse**

Avant de porter secours, je dois m'assurer…

a. …de ne pas me mettre en danger.

b. …qu'il y ait de la place pour garer l'ambulance.

c. …d'avoir une trousse de secours avec moi.

**2. Indique le numéro de téléphone à composer.**

Urgences médicales en Suisse : __________________

**3. Entoure les bonnes réponses**

Lorsque j'appelle les secours, j'informe au téléphone...

*Ce que j'ai déjà fait	Le lieu/adresse	Ce qu'il s'est passé*

**4. Coche si c'est vrai ou faux**

Pour vérifier si une personne respire normalement,...

Vrai	Faux

je dois libérer les voies respiratoires	🞏	🞏

j'observe, je sens et j'écoute pendant 5 à 10 secondes	🞏	🞏

je regarde si elle a l'air bizarre ou si elle est bleue	🞏	🞏

je regarde uniquement si la poitrine se soulève	🞏	🞏

**5. Coche si c'est vrai ou faux**

Je décide de mettre une personne en PLS, parce que :

Vrai	Faux

la personne crie fort et semble souffrir.	🞏	🞏

la personne est inconsciente, mais respire normalement.	🞏	🞏

la personne est consciente et respire profondément.	🞏	🞏

la personne est inconsciente et ne respire pas normalement.	🞏	🞏

Il y a encore 3 questions derrière ☺ **🡪**

**6. Coche si c'est vrai ou faux**

Comment reconnais-tu une personne en arrêt cardiaque ?

Vrai	Faux

La personne crie fort et semble souffrir.	🞏	🞏

La personne est inconsciente, mais respire normalement.	🞏	🞏

La personne est consciente et respire profondément.	🞏	🞏

La personne est inconsciente et ne respire pas normalement.	🞏	🞏

**7. Entoure la bonne réponse**

Si je ne suis pas sûr qu'une personne est en arrêt cardiaque...

a. ...je commence quand même la réanimation.
b. ...je place la personne en position latérale de sécurité (PLS) et j’attends l’arrivée des secours.
c. ...j'appelle les secours et leur demande conseil.

**8. Coche la réponse qui te correspond**

Maintenant, je me sens capable d'appeler le numéro d'ugence :

OUI	🞏	PEUT-ETRE	🞏	NON	🞏
