# Supplementary material for: Recruiting and Training Junior Medical Students as Basic Life Support Instructors for Primary School Students: Prospective Pilot Implementation Study
Source: JMIR Form Res. 2026 Sep 3;10:e100385. doi: 10.2196/100385 (PMC13541228; doi:10.2196/100385)
Supplement: Supplementary file 2 [file formative-v10-e100385-s003.docx]

|   Office cantonal de l'enfance et de la jeunesse | republique et canton de geneve  Département de l'instruction publique, de la formation et de la jeunesse  Office cantonal de l'enfance et de la jeunesse  Service de santé de l'enfance et de la jeunesse | | |  |
| --- | --- | --- | --- | --- |
|  |  |  |  | |
|  | DIP - SSEJ  Direction  Rue des Glacis-de-Rive 11  1207 Genève      Réf : L 861/SGT/tfn |  | Aux parents des élèves des classes de 7P du projet pilote « Se former aux gestes qui sauvent ! »    Genève, le 19 mars 2025 | |

**Objet :	Information et consentement concernant le projet pilote d'éducation à la santé « Se former aux gestes qui sauvent ! »**

Madame, Monsieur

Dans le cadre du déploiement progressif de nouveau cours d'éducation à la santé dans les écoles du DIP intitulé Parcours santé de l'élève, la classe de votre enfant bénéficiera prochainement d'un cours sur les premiers secours (appeler à l'aide, évaluer la respiration et la conscience, appeler le 144, position latérale de sécurité, introduction au massage cardiaque sur des mini mannequin prévu pour les enfants). Il est d’une durée de deux périodes (1h30), sera réalisé en classe en présence de l’enseignant ou enseignante de votre enfant.

Ce cours est organisé par le Service de santé de l’enfance et de la jeunesse du DIP en collaboration avec la Faculté de Médecine de l'Université de Genève et la Brigade Sanitaire Cantonale des Hôpitaux Universitaires de Genève.

Nous sollicitons **votre consentement** pour la soumission d’un court questionnaire d’évaluation des contenus du cours à votre enfant à la fin du cours (durée 5 min) dans le but de:

1. Vérifier les connaissances acquises, conformément aux recommandations scientifiques dans le domaine.
2. Évaluer leurs compréhensions des messages clés tout en mesurant l'efficacité de ce cours.

L'analyse anonymisée des résultats du questionnaire permettra d'améliorer les contenus des cours de sensibilisations aux premiers secours dans le but de progressivement le généraliser pour toutes les classes de 7P.

Les responsables du projet, présents en classe expliqueront de vive voix aux élèves que leur participation est **facultative et volontaire**. Les enfants sont libres de refuser d'y répondre ou d'interrompre leur participation à tout moment, sans justification. Il sera également précisé que le questionnaire n'est **pas une évaluation notée**, et qu'**aucune donnée personnelle** permettant d'identifier les élèves ne sera recueillie.

En vous remerciant, pour l’attention portée à la présente, nous vous adressons, Madame, Monsieur, nos meilleures salutations.

**Pascal Freydier**

Directeur SSEJ DIP

11 Glacis de Rive

1207 Genève

Tél. 022 546 41 00

**Talon réponse à remettre à l’enseignant ou l’enseignante de votre enfant**

Nom et prénom de l’élève : __________________________________________

🞏	J'ai pris connaissance de ce qui précède. Je permets à mon enfant – sous réserve de son consentement – de répondre au questionnaire tout en sachant que ses données anonymes puissent être utilisées à des fins de recherche scientifique.

🞏	J'ai pris connaissance de ce qui précède. Je ne souhaite pas que les données anonymes provenant de mon enfant soient utilisées à des fins de recherche scientifique.

Date : ________________________	Signature d'un parent : ___________________
